# Advanced Strategies for Upgrading Raw Biogas into High-Quality Biomethane for Domestic Applications

**DOI:** 10.3390/bioengineering13050543

**Published:** 2026-05-09

**Authors:** Reckson Kamusoko, Patrick Mukumba

**Affiliations:** Computational Science Department, Faculty of Science and Agriculture, University of Fort Hare, P. Bag X1314, Alice 5700, South Africa; pmukumba@ufh.ac.za

**Keywords:** anaerobic digestion, biomethane, fossil fuels, purification, raw biogas

## Abstract

Biogas produced from the anaerobic digestion of organic matter holds much promise as a renewable energy source for decentralized systems. However, raw biogas contains substantial volumes of carbon dioxide, hydrogen sulfide, water vapor, and other trace impurities. These impurities can reduce the calorific value of biogas and limit its direct use for household energy needs. Purifying biogas to high-grade biomethane (≥95%) is therefore important to improve methane (CH_4_) content and combustion characteristics. This is a guarantee of its safe utilization in domestic appliances, including cooking, heating, lighting, and electricity generation. This article reviews and evaluates novel approaches for upgrading raw biogas into high-purity biomethane that can offset natural gas in domestic applications. It further examines recent developments in conventional and innovative upgrading technologies such as water scrubbing, chemical scrubbing, pressure swing adsorption, membrane separation, cryogenic separation, and biological upgrading. Particular emphasis is placed on low-cost and small-scale solutions suitable for off-grid or mini-grid rural energy systems. Moreover, the role of process optimization, intelligent monitoring, and data-driven control methods in increasing CH_4_ recovery and process efficiency is discussed. Despite their relatively high capital costs and energy needs, conventional technologies such as water scrubbing, pressure swing adsorption, and membrane technology continue to dominate biogas purification systems. The findings show that coupling advanced separation technologies, including cryogenic separation, biological upgrading, and hybrid technologies, with optimized process control can significantly improve CH_4_ purity, save energy use, and enhance the overall consistency of biogas purification systems. These innovative strategies have strong potential to promote the full-scale adoption of biomethane as a clean, sustainable, and affordable energy source for decentralized applications, particularly in the developing world.

## 1. Introduction

In recent decades, the search for alternative fuel sources has intensified due to depleting fossil fuels, rapid population growth, the rise in fuel prices, and increasing environmental degradation concerns [1,2,3]. Bioenergy has emerged as a promising solution for reducing carbon footprints and mitigating environmental impacts [2]. Among the different forms of bioenergy, biogas is gaining traction as a renewable substitute for conventional fossil fuels. Its growing popularity is largely attributed to the economic viability of its production process. At present, biogas contributes only a small fraction to the world energy mix. In 2023, the total global biogas potential was estimated at approximately 40 billion cubic meters of natural gas equivalent. However, the global biogas production is increasing rapidly and is predicted to rise by around 22% between 2025 and 2030, supported by more than 50 new policies introduced in 2022. The bulk of biogas production comes from Europe, the United States, and China [4]. Generally, raw biogas is used as a source of heat and electricity. However, it has relatively low calorific value of about 20–25 MJ/m^3^ compared to natural gas, which restricts its direct applications [5].

Biogas is generated by the anaerobic digestion (AD) of organic matter, including manure, municipal solid waste, crop residues, and food waste [6]. AD is a multistep process performed by bacteria consortia, and involves four main stages: hydrolysis, acidogenesis, acetogenesis, and methanogenesis [3,5]. Biogas is mainly made up of methane (CH_4_) and carbon dioxide (CO_2_) [7]. However, it also contains impurities such as water vapor (H_2_O), hydrogen sulfide (H_2_S), and trace gases. The presence of CO_2_ and H_2_O lowers the heating capacity of biogas. H_2_S is highly corrosive to metallic biogas appliances and their supply systems [8,9]. For this reason, biogas purification or upgrading is essential to remove these impurities and meet the natural gas quality standards.

Biogas upgrading can increase CH_4_ concentration to more than 95%, producing biomethane with properties comparable to natural gas. Consequently, purified CH_4_ can compete with conventional fuels like gasoline, petrodiesel, fuelwood, hydrogen (H_2_), and natural gas in the global energy market [9,10]. However, the biomethane quality may vary depending on its intended final use. For example, most European countries regulate gas quality for grid injection under the EN 16723-2:2018 guidelines [11]. According to these specifications, biogas should meet stringent composition criteria, including ≥90% CH_4_, ≤2% CO_2_, ≤1% O_2_, and absolutely no H_2_S [12].

Biomethane is a renewable energy carrier that can contribute significantly to the development of a circular economy model. Purified biogas can be used as a vehicle fuel or injected into natural gas pipeline systems for heating and other energy applications [13,14]. The use of upgraded biogas as a transportation fuel is anticipated to increase by roughly 25% by 2050 [2]. Several technologies have been proposed to upgrade raw biogas into high-quality biomethane, with most work focusing on the removal of CO_2_ and H_2_S impurities. Biogas upgrading strategies can generally be grouped into physical, chemical, biological and combined processes [9,13]. While many of these technologies are still under research and development, commercially available methods include adsorption, absorption, membrane separation and cryogenic separation [2].

In Europe, the total installed capacity of biomethane plants increased by 15%, from 1309 plants in 2022 to 1510 in 2023 [15]. Germany and France lead in biogas upgrading infrastructure, with over 232 and 131 upgrading plants, respectively. France has a goal to generate 8 TWh of electricity from upgraded biogas by 2023 [2]. In Sweden, upgraded biogas is widely used as a transportation fuel, powering a large proportion of vehicles [4]. The country is expected to achieve 100% reliance on biomethane as a vehicular fuel by 2028 [2]. Ongoing research efforts are concentrating on optimizing and enhancing the overall efficiency and cost-effectiveness of biogas upgrading facilities [13].

Monitoring and control strategies have been developed in order to increase the CH_4_ concentration during biogas upgrading. The emphasis is on optimizing and stabilizing operating parameters such as pressure, temperature, flow rates, pH, and reagent concentration. Effective process control ensures that initial capital, maintenance, and operational costs are minimized [16]. Biogas upgrading systems can be monitored using off-line devices, on-line sensors, mathematical models, and advanced technologies to track the quality of gas (e.g., CH_4_, CO_2_, and H_2_S levels) and regulate process conditions [17,18,19,20]. This permits real-time modifications to preserve stable and high-quality biomethane production. Key monitoring components comprise sensors for multiple variables, viz., pH, volatile fatty acids (VFAs), temperature, and gas composition, together with advanced analytical tools. These components can be combined with automated control systems and process analytical technology (PAT) to improve performance, stability and overall biomethane yield [18,19,21].

Despite the substantial advances made in biogas purification, several technical bottlenecks continue to hinder their full-scale adoption. Numerous upgrading systems face steep running costs, advanced process regulation, and high energy demand, which can influence their overall economic viability. More so, variabilities in raw biogas mix, process conditions, and impurities in the mix can affect the efficacy and stability of the purification methods.

Even though traditional processes, including absorption, adsorption, and membrane separation methods, have shown promising results, additional adjustments are obligatory to improve CH_4_ yield, lower energy input, and boost process feasibility and sustainability. As a result, continued research efforts are pertinent in designing cutting-edge and competent approaches for converting raw biogas into high-purity biomethane. The overarching aim is to review and analyze recent advancements in biogas purification and explore innovative technologies that can improve biomethane generation for sustainable domestic energy applications.

## 2. Principles of Anaerobic Digestion

**Figure 1:**
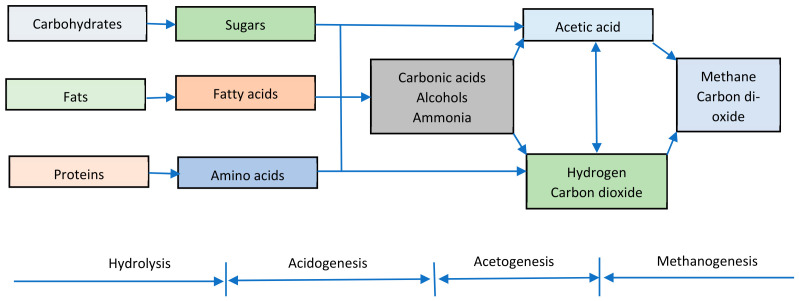
The anaerobic digestion process [25].

AD is a four-stage process, involving hydrolysis, acidogenesis, acetogenesis, and methanogenesis [22]. A process flow chart for the various stages of AD is shown in Figure 1. AD is a mature process, whereby organic matter is anaerobically degraded into biogas using microbial consortia. A variety of biomass resources such as animal manure, agricultural residues, dedicated crops, sewage sludge, and municipal solid waste are suitable feedstocks for biogas production [23]. Digestate is the waste by-product from the degradation of organic matter. It is a nutrient-rich bioresource for fertilizer production [24].

Hydrolysis is the first step in the AD of organic materials. It involves the use of hydrolytic bacteria to break down complex organic compounds into simple fermentable molecules. In acidogenesis, acidogenic bacteria are utilized to ferment hydrolytic products into VFAs, alcohols, ketones, CO_2_, ammonia (NH_3_), H_2_, and H_2_S [25]. Acetogenesis is the bioconversion of VFAs, alcohols, and other intermediary products into acetic acid, H_2_, and CO_2_. This process is carried out by a group of acetogenic bacteria. The final products of the AD process are CH_4_ and CO_2_, produced during the methanogenesis stage. Here, acetate-, CO_2_-, and H_2_-consuming bacteria utilize simple substrates, such as acetic acid, H_2_, and C_1_ compounds. In addition, methylotrophic methanogens can synthesize CH_4_ using methylated compounds as substrates. The primary sources of CH_4_-producing bacteria are swamps, lakes, marine sediments, ruminant guts, and artificial environmental conditions [25].

It is critical to monitor and control environmental variables, mostly temperature and pH, that affect the methanogenic step of the AD process. The most favorable pH for methanogenic bacteria ranges from 6.6 to 7.8. The optimal temperatures for psychrophiles, mesophiles, thermophiles and hyperthermophiles are around 18 °C, 37 °C, 55 °C and 65 °C, respectively [5].

## 3. Composition and Properties of Biogas

Biogas is a mixture of various gases, with differing percentage compositions depending on digester temperature and feedstock type (Table 1). The main component of biogas is CH_4_, which makes up about 55–70% of the total gas and is a valuable source of energy. Biogas also consists of around 35–40% CO_2_ by volume [26]. In addition, it contains 0–10% (*v*/*v*) of nitrogen (N_2_) and oxygen (O_2_) gases, as well as trace amounts of other impurities such as H_2_, H_2_S, H_2_O, NH_3_, carbon monoxide, and volatile organic compounds [27]. Some of these gases, especially H_2_S, H_2_O and CO_2_, reduce the energy quality of biogas and can pose challenges to its energetic potential and can damage biogas appliances. Therefore, it is critical to remove these gases to ensure a high-quality biogas value chain [26].

Biogas is colorless and tasteless and has a characteristic rotten-egg odor due to the presence of H_2_S. It is a greenhouse gas and can cause about 25 times more greenhouse effect than CO_2_. Upon combustion, biogas produces a characteristic blue flame at around 800 °C. It can ignite rapidly when mixed with air at a ratio of 1:20. An optimum pressure range of 5–20 cm water column has been reported for cooking with biogas. Approximately 5500–6500 kcal of heat can be generated from burning one cubic meter of biogas. This can power a one-horsepower internal combustion engine for about two hours. One cubic meter of biogas corresponds to about 0.4 kg, 0.6 kg and 0.8 kg of petrodiesel, oil, and coal, respectively [26].

## 4. Biogas Upgrading Technologies

Biogas is considered a renewable source of energy that can replace natural gas in heat and power generation and as a vehicular fuel [6]. However, the presence of impurities adversely affects metallic biogas equipment and limits its energetic potential [29]. Furthermore, impurities may impose serious health threats like respiratory ailments, appetite loss, etc., on end-users. For example, H_2_S concentration in the range of 150–2000 ppm has been reported to cause eye damage, fatigue, respiratory challenges, immediate collapse, and even death. Approximately 10–15 ppm of H_2_S is generally regarded as the safe exposure limit. High CO_2_ levels reduce the calorific value of biogas and freeze the pipeline system [6]. This calls for purification and concentration of biogas to enhance the heating efficiency and remove impurities.

**Figure 2:**
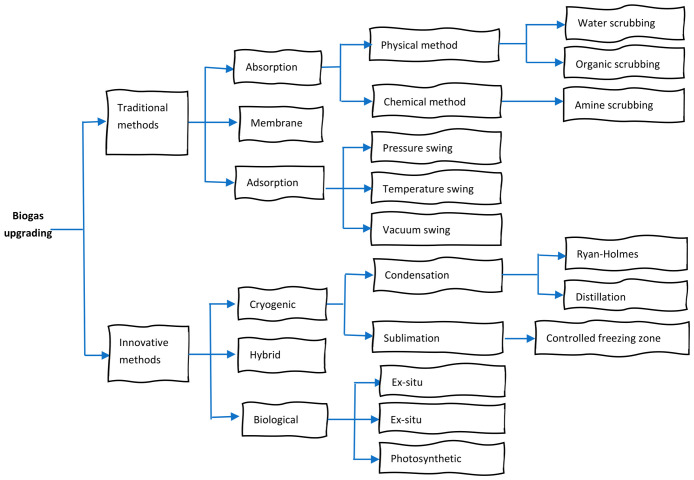
Technologies for biogas upgrading [32].

Biogas upgrading is a multistep technology, involving the removal of CO_2_ and other impurities, as well as drying and compressing the gas. The final pure product is called biomethane or bionatural gas [13,30]. Biogas purification is broadly classified into traditional/conventional and innovative technologies, which include physical, chemical, biological, and combined processes (Figure 2). Traditional biogas upgrading primarily focuses on the removal of CO_2_ to improve the CH_4_ content and the calorific value of the gas. In contrast, emerging and innovative upgrading technologies aim for a more comprehensive purification approach. These modern systems integrate the concurrent removal of CO_2_, H_2_S, and other gaseous impurities in a single or integrated process [31].

The mechanisms, merits and demerits of biogas upgrading technologies are presented in Table 2. Generally, conventional biogas upgrading is a mature and commercially established technology. However, challenges persist in terms of cost, energy requirements, and scalability, particularly for decentralized solutions. Emphasis is being placed on new technologies such as cryogenic separation, in situ upgrading, hydrate separations and biological treatment. Most of this work focuses on laboratory and pilot phases to remove moisture (drying), H_2_S (desulphurization) and CO_2_ (enhance heating capacity). It is important to transfer technology from small-scale research towards fully commercialized projects [13].

### 4.1. Physical Processes

Physical methods for biogas purification remove unwanted substances from raw biogas using physical separation processes. These technologies primarily target impurities such as H_2_O, CO_2_, and H_2_S, without altering the chemical structure of the gas. The purpose is to improve the CH_4_ content and the overall energy quality of biogas. Several physical separation technologies are widely reported in the extant literature [13,34]. These include absorption, adsorption, water scrubbing, cryogenic separation, and membrane separation [5,29]. Physical methods are widely used due to their simplicity and ease of operation.

#### 4.1.1. Water Scrubbing

**Figure 3:**
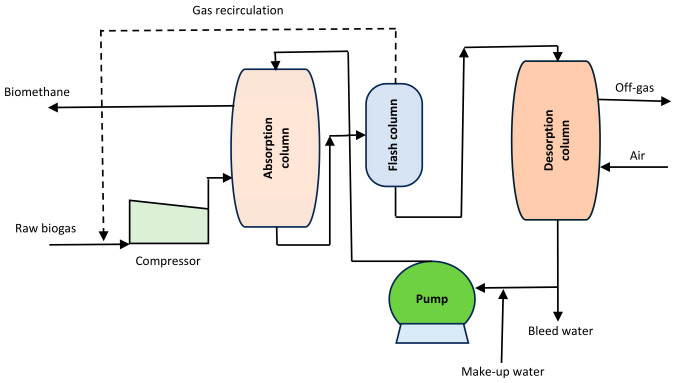
Water scrubbing system for biogas upgrading [34].

Water scrubbing is viewed as a simple, mature, and affordable technology to separate CO_2_ and H_2_S from biogas [13]. Using this method, Khan et al. [33] claimed that more than 97% pure CH_4_ can be recovered from the biogas. This mechanism is utilized by around 41% of the biogas purification plants in the world. A schematic presentation of the pressurized water scrubbing process is shown in Figure 3. It is based on Henry’s law, which affirms that the volume of any dissolved gas is directly related to its partial pressure at a steady temperature. As such, it is assumed that CO_2_ is 25% more soluble in water than CH_4_ at 25 °C [3]. H_2_S is removed from the system due to its higher solubility in water than CO_2_. The effluent enriched with CO_2_ or H_2_S and trace amounts of CH_4_ is recovered from the scrubber and recycled into the system [33].

The efficiency of water scrubbers may vary depending on the packaging material used. For example, Noorain et al. [35] proposed a new water scrubbing technique and replaced traditional packaging materials with sponge carriers. Their study achieved over 90% CH_4_ purity, with no detectable H_2_S from biogas initially containing 60% CH_4_. In another study in Ghana, the designed water scrubber removed about 93% (*v*/*v*) of the CO_2_ from raw biogas [36], whereas water scrubbing at gas pressure of 400 kPa and water flow rate of 0.15 L/s removed about 99.5% CO_2_ and produced 38% more CH_4_ [37].

#### 4.1.2. Membrane Separation

**Figure 4:**
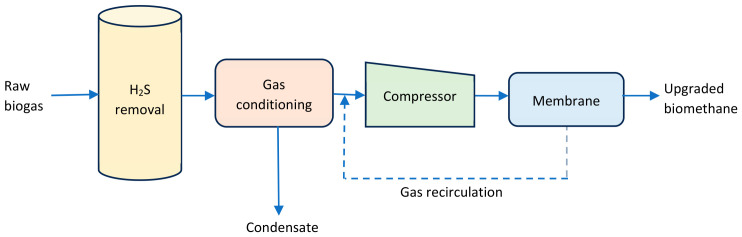
Schematic diagram of membrane gas permeation [38].

The membrane technology is based on separation or permeation of biogas through a partially permeable membrane. The process flow chart of the membrane separation technique is depicted in Figure 4. Membrane gas permeation occurs at low pressure of around 100 kPa or high pressure in the range of 2000–3600 kPa [13]. There are several types of membranes that can allow permeates (e.g., CO_2_, H_2_S, and H_2_O) to pass through while retaining and producing high-purity bionatural gas [29]. These include polymeric materials, inorganic membranes, and mixed matrix membranes. Typical examples of polymeric membranes include cellulose acetate, cellulose triacetate, polyimides, polyetherimide, polyamide, brominated polycarbonate, and polymethyl pentene [29]. Inorganic membranes such as glass, ceramic, metallic, zeolite, and carbon are more stable, efficient and durable than polymeric membranes. However, they are costly and difficult to fabricate [38]. The final CH_4_ purity of membrane separation falls within the range of 90–97% [13].

In the Czech Republic, membrane purification was evaluated at pilot scale using ideal biogas and the results achieved a CH_4_ purity of 95% [39]. Marsico et al. [40] designed an integrated membrane gas permeation system and concluded that more than 97.5% CH_4_ gas from this system could be directly injected into the natural gas grid. Purifying biogas from sewage plants through the DDR-type zeolite membrane indicated CH_4_ purity and CO_2_ recovery of 90% and >97%, respectively [41]. Compared to other upgrading technologies, membrane separation is a low-cost method that is easy to operate and scale up [38].

#### 4.1.3. Cryogenic Separation

Cryogenic separation is the liquefaction or solidification of various gaseous components at varying pressure and temperature conditions [28,35]. There are several approaches to cryogen separation, including cryogenic distillation, anti-sublimation and controlled-freeze-zone processes. Cryogenic distillation is more advantageous over the other methods. It produces high CH_4_ volumes with a high CO_2_ recovery [42].

**Figure 5:**
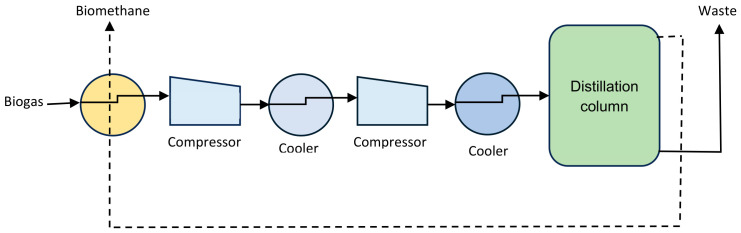
Schematic chart of the cryogenic separation [33].

Figure 5 represents a flow diagram of the cryogenic separation via the distillation method. In this process, CO_2_ is removed from biogas because its boiling point (−78.2 °C) is higher than that of CH_4_ (−161.5 °C) at 100 kPa pressure [38]. Biogas components like H_2_O, H_2_S, siloxanes and halogens must be removed before CO_2_ separation to prevent freezing and clogging of the system [29]. Cooling biogas at low temperatures ensures condensation or sublimation of CO_2_ and other impurities, as well as collection of CH_4_ in the gaseous phase. Cryogenic separation can generate up to 99% CH_4_ purity [6].

In a study by Yousef et al. [43], a mixture of biogas containing 60% CH_4_ and 40% CO_2_ was separated using one distillation column. The results revealed biomethane purity and CO_2_ recovery of 94.5% and 99.7%, respectively, along with specific energy consumption of 0.26 kWh/Nm^3^. Knapik et al. [44] used the cryogenic method to separate liquified CO_2_ from flue gases generated by oxy-fuel combustion. A total of 83.07% CO_2_ and 99.17% CH_4_ purity were recovered from this novel system. This consumed about 0.10 kWh/Nm^3^ CO_2_ of energy.

However, cryogenic separation is costly due to high energy needed for cooling and freezing the CO_2_. The energy is used by upgrading equipment such as distillation columns, heat exchangers, turbines, and compressors. It is suggested to concentrate CO_2_ in the biogas to minimize energy use and improve the economic viability of the cryogenic separation process [38].

#### 4.1.4. Pressure Swing Adsorption

**Figure 6:**
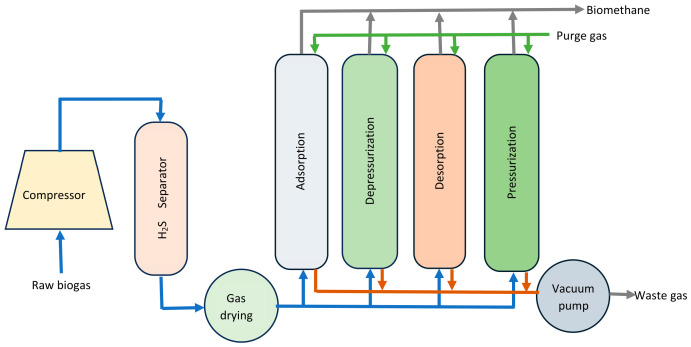
Biogas purification by pressure swing adsorption [48].

Pressure swing adsorption is one of the most promising methods in the world, contributing approximately 21% to the biogas upgrading technologies [29]. This method exploits porous adsorbents to selectively adsorb and desorb impurities in biogas at different operating pressures [38]. Adsorbents such as activated carbon, silica gel, activated alumina, zeolite, and polymeric adsorbents are readily available in the global market. A schematic diagram of biogas purification using the pressure swing adsorption process is shown in Figure 6. In this process, compressed gases adhere to solid surfaces and low pressure causes the release of purified gas [6]. Pressure swing adsorption depends on the molecular size of gas particles and the adsorbent affinity. Adsorbents have an average pore size of 0.37 nm, and selectively retain CO_2_ (0.34 nm) and exclude CH_4_ (0.38 nm) [29].

Pressure swing adsorption is preferred to other adsorption technologies. This can be attributed to its low energy and equipment needs. More so, biomethane recovery in the range of 95–99% can be attained using this technology. As an exemplar, Augelletti et al. [45] tested two pressure swing adsorption units based on zeolite 5A adsorbent for their potential to purify biogas. Over 99% of the biomethane was recovered from this system while utilizing 1250 KJ/kg CH_4_ of energy. In Taiwan, a full-scale pressure swing adsorption system accomplished about 99.28% CH_4_ purity with 91.44% recovery and H_2_S purity of 0.015 ppm. This system consumed 860 KJ/kg-CH_4_ of power [46]. According to the model proposed by Santos et al. [47], using pressure swing adsorption to upgrade a biogas stream of 500 Nm^3^/day can produce more than 99% pure CH_4_ with 85% recovery and energy consumption of 1.487 × 10^−3^ kWh/Nm^3^ of the produced bionatural gas. Contrarily, it is difficult to recycle adsorbents due to permanent adsorption of H_2_S to the molecular sieves. Therefore, it is recommended to dry the gas and separate H_2_S before adsorbing the CO_2_ [34].

### 4.2. Biological Processes

Biological upgrading is a suitable alternative that can replace physical and chemical methods in biogas purification [34]. It is an environmentally benign technology that does not utilize solid adsorbent materials, chemicals and membranes to upgrade biogas [3]. Common biological systems that can transform CO_2_ into beneficial products include chemoautotrophic upgrading, photoautotrophic purification, H_2_S removal, biofiltration, microaeration desulphurization, and so on [3]. Microorganisms are central to the transformation of H_2_S in biological upgrading systems. They utilize H_2_S as an energy source for growth [31].

#### 4.2.1. Biofiltration

**Figure 7:**
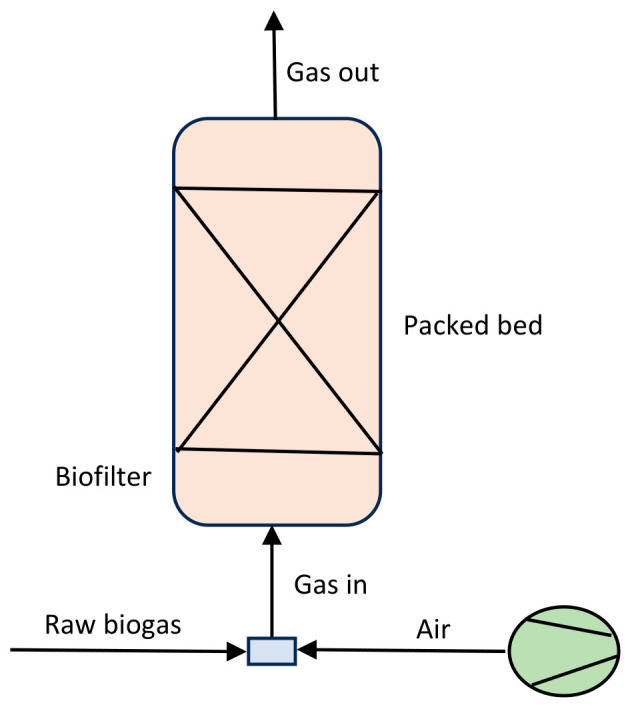
Schematic presentation of biofiltration system [50].

Biofiltration exploits biofilms in a fixed-bed bioreactor to treat raw biogas. A generalized scheme of biofiltration for biogas upgrading is shown in Figure 7. The system utilizes either biofilters or biotrickling filters depending on the type of bed material. These consist of organic (e.g., peat, compost, and wood bark) and inorganic inert carriers (e.g., ceramic, pozzolan, and marble), respectively [31,49]. The basic principle is the flow of untreated biogas through a column packed with permeable material and its diffusion into an immobilized biofilm, where H_2_S is oxidized. An O_2_ concentration of 5–10% (*v*/*v*) is directly injected into the system. The packed bed contains a large surface area to support the growth of biofilm on extracellular polymeric matrix. It serves as a source of nutrients or a nutrient solution that can be intermittently supplied from the top of the system [50].

Biofilms are composed of specialized bacteria (chemolithoautotrophs and other microorganisms) that can utilize H_2_S as an energy source to generate sulfur or sulfates. They possess the advantage of immobilization within a microbial community, where communal and synergistic interactions, homeostasis, and autopoiesis collectively improve microbial stability, resilience, and overall functional performance [51,52].

Biofiltration can result in 100% H_2_S separation, 96% C0_2_ removal and 96% CH_4_ purity or more [6]. Porte et al. [51] carried out a study to investigate the process performance and microbiome structure of lab-scale thermophilic biotrickling filters for biomethanation of CO_2_ and H_2_S. The produced gas exhibited high-quality biomethane with CH_4_ volumes exceeding 97%, which is analogous to the quality of natural gas. Furthermore, the microbiome analysis showed that bacteria such as *Methanothermobacter* sp., *Clostridia*, and acetate-degrading bacteria were dominant in the biofilm and other components of the biogas upgrading system. On the other hand, the integrated biofilter system containing a packed bed of soil, biochar and the moss plant revealed enhanced biomethane quality. A sorption capacity of 11 g S and recovery efficiencies of 68% CO_2_, 72% NH_3_, and 93% H_2_S were reported from this study [53].

Factors such as packaging or filter bed materials, inoculum type, biofilm characteristics and process conditions play a critical role in determining the efficiency of biofiltration systems [31]. Therefore, continuous monitoring and proper management of these parameters are essential to ensure stable operation, high removal efficiencies, and long-term performance.

#### 4.2.2. Chemoautotropic Processes

**Figure 8:**
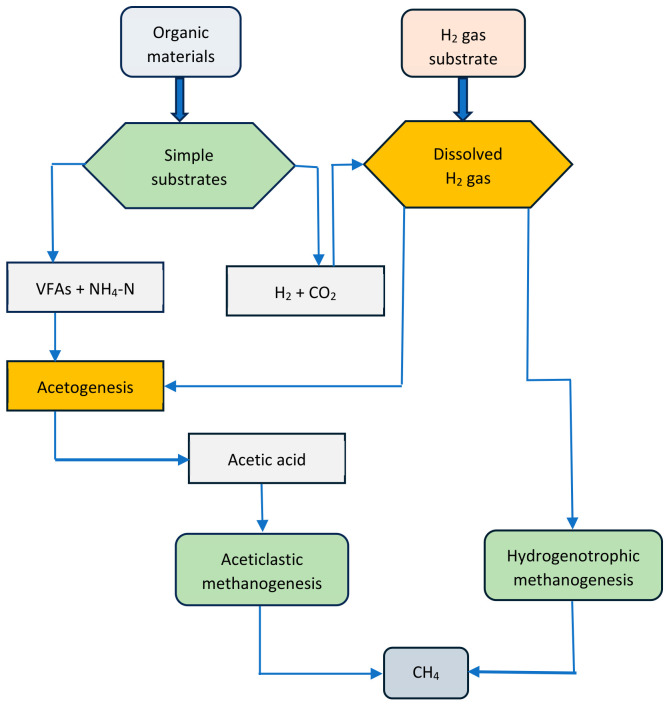
Flow chart of the acetate-oxidizing bacteria-assisted metabolic pathway utilizing hydrogen substrate and hydrogenotrophic methanogens [56].

Chemoautotropic upgrading involves the use of hydrogenotrophic methanogens to transform CO_2_ in biogas into high-grade CH_4_ gas (Figure 8). In this process, CO_2_ reacts with gaseous H_2_ to produce CH_4_ and H_2_O. The primary source of H_2_ is the electrolysis HCl-H_2_O solution [48,49]. CO_2_ acts as the carbon source and electron acceptor and H_2_ serves as the electron donor (Equation (1)). The produced H_2_ cannot be stored due to low energy density per unit volume [48]. Therefore, it is quickly utilized as a catalyst to reduce CO_2_ to biomethane. By-products like acetate, ethanol, and other aqueous solutions are released during this process [54].

(1) $${4H}_{2}+{CO}_{2}\to {CH}_{4}+{2H}_{2}O$$

Various organic compounds such as acetate, methanol, and other intermediates can also act as substrates for specific hydrogenotrophic methanogens. Hydrogenotrophic methanogens have also been found to tolerate high NH_3_ conditions of approximately 5.5 × 10^3^ mg/m^3^ [10]. *Methanospirillum*, *Methanococcus*, *Methanosarcina*, *Methanobacterium*, *Methanoculleus,* and *Methanothermobacter* are typical examples of hydrogenotrophic methanogens [10,49]. Rao et al. [55] evaluated the potential for CO_2_ removal in a novel three-phase biogas upgrading system supplied with H_2_ through a gas-permeable membrane. The produced biogas had high CH_4_ content of up to 95%. Hydrogenotrophic members of Methanobacteriaceae, with a relative abundance of 71.9%, were identified as the most prevalent microorganisms within the biofilm. Hydrogenotrophic-based biogas upgrading is simple and has high technological potential [56].

#### 4.2.3. Microaerobic Desulfurization

Microaerobic desulfurization combines microaeration and oxygenation to promote the growth of sulfide-oxidizing bacteria (SOB) [3]. It is a simple, low-cost, efficient, and reliable method to remove H_2_S from biogas by transforming sulfide oxide into elemental sulfur (S^0^) or sulfate using SOB. The outcome is a function of SOB species used, their functional genes, and the O_2_ dosage [57]. As shown in Equation (2), the process involves the partial oxidation of sulfides in anaerobic bioreactors. Elemental sulfur is precipitated and removed from the bioreactor in a mixture of digested sludge [58,59,60]. It is critical to monitor the amount of O_2_ supply to maintain the anaerobic conditions in bioreactors. Injecting air directly into the reactor unit is discouraged because it often dilutes the gas with N_2_ and O_2_, and may also promote the accumulation of elemental sulfur that can block the biogas transmission line. This can be avoided by using a membrane packed with a biofilm to separate air from the biogas stream [59].

(2) $${2H}_{2}S+O_{2}\to {2S}^{0}+{2H}_{2}O$$

Microaeration is a potent strategy for separating H_2_S from the AD of sludge and wastewater. Besides desulfurization, several other benefits of microaeration associated with improved CH_4_ recovery were reported by Khaskheli et al. [60]. In central Europe, an investigation of seven full-scale microaerobic reactors treating municipal wastewater showed a desulfurization efficiency ranging from 74% to 99%. In addition, the degradation of chemical oxygen demand and volatile suspended solids were significantly increased [58]. The microaerobic treatment of 5000 ppm H_2_S, at an air injection rate of 0.6–1.0% O_2_ and a biogas flow rate in the range of 0.9–1.8 L/h-L inoculum mix, achieved a desulfurization efficiency of 99–100% [61].

#### 4.2.4. Bioscrubbing

**Figure 9:**
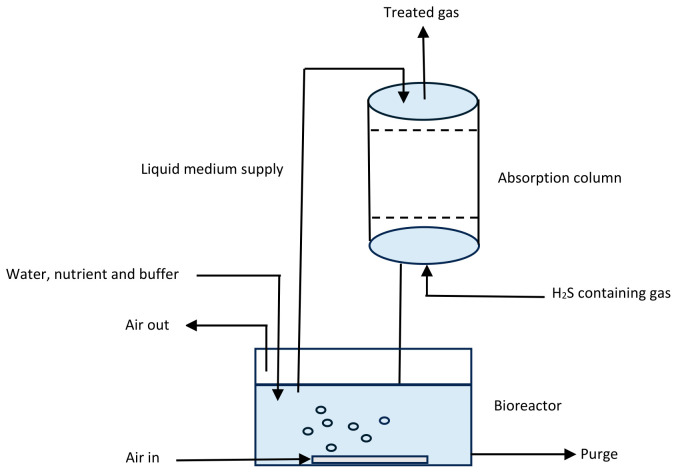
An overview of the removal of hydrogen sulfide using the bioscrubbing technology [31].

Bioscrubbing is a two-component system that uses an absorption tower and a bioreactor unit to decontaminate raw biogas (Figure 9). Bioscrubbers are mostly applied to purify gases contaminated with water-soluble compounds such as NH_3_ and H_2_S. The function of the absorption tower is to remove pollutants, mainly H_2_S, dissolved in water [32,52], whereas the bioreactor harbors specific microflora that can utilize CO_2_ as a carbon source to turn H_2_S into sulfur and sulphates. These microorganisms include *Thiobacillus*, *Thiomonas*, *Paracoccus*, *Acidithiobacillus*, *Sulfurimonas,* and *Halothiobacillus*. Biomass and H_2_O are produced as by-products of this bioreactor system. It is important to regularly monitor pH (8.5–9.0) and other process variables, and remove the sulfur-rich waste from the system. Hence, a settler is incorporated into the system to separate biomass waste and sulfur from the liquid phase, allowing the clarified fluid to be recycled back into the absorption column [28,52].

A pilot study was conducted in the absence of O_2_, to evaluate the prolonged performance of a bioscrubber for the desulfurization of landfill biogas. The maximum H_2_S recovery efficiency of 97% was achieved under operating conditions, over a period of 61 weeks. Using metagenomic and bioinformatics analysis, *Thioalkalispira-Sulfurivermis* with over 18% abundance was observed to be the most abundant genera in the upgrading system [62]. Bioscrubbers are able to tolerate several environmental conditions with high removal efficiency of water-soluble impurities. Nonetheless, the system is difficult to maintain and run and may produce secondary pollutants from the liquid waste stream [31].

#### 4.2.5. Photosynthetic CO_2_ Bio-Fixation

It is possible to remove CO_2_ and H_2_S in photobioreactors using anaerobic phototrophs in the presence light and CO_2_ [9,10]. CO_2_ fixation by photosynthetic organisms offers a highly sustainable and biologically driven approach to biogas purification. Photoautotrophic organisms, including microalgae and cyanobacteria, can metabolize CO_2_ as a source of carbon, transforming it into organic compounds. The use of microalgae can be ascribed to rapid growth rates, ability to utilize waste nutrients, and capacity to tolerate diverse ecological conditions [10]. Species such as *Spirulina, Chlorella*, and *Arthrospira* have been reported to possess high photosynthetic activity for gas purification [48]. In this process, raw biogas is initially introduced into the photobioreactor system. The mechanism of CO_2_ concentration is utilized to lower CO_2_ levels and augment the amount of atmospheric O_2_. The system can also remove trace amounts of H_2_S, either biologically using the SOB that co-exist in the reactors or chemically by reacting with waste O_2_ released by microalgae [10]. Solar energy, nutrients, CO_2_ from biogas, and water are the essential requirements for microbial growth. This exothermic process produces biomethane with over 97% purity, as well as by-products such as biomass and O_2_ gas. The final CH_4_ gas contains approximately 2–6% CO_2_. The produced biomass can be utilized as digester feed to generate more CH_4_ [3,9].

In Mexico, a study was conducted to upgrade biogas from swine waste into pure biomethane using a microalgae–bacterial photobioreactor system. The purified biogas was found to contain no more than 6.8% CO_2_ and up to 98.9% recovery efficiencies for H_2_S [7]. A pilot-scale biogas upgrade using an algal–bacterial-based photobioreactor system produced CO_2_ and CH_4_ concentrations of 0.7–11.9% and 85.2–97.9%, respectively. Also noteworthy was the maximum biomass productivity of 22.5 g/m^2^ per day [8]. In yet another study, the photosynthetic-driven biogas purification was successfully validated at a semi-industrial scale using an algal–bacterial photobioreactor. In this system, CO_2_ and H_2_S recovery efficiencies of 90% and 100%, respectively, were reported under a liquid-to-biogas ratio of 3.5. In addition, the highest CH_4_ concentration of 90% was detected. This biomethane output was mainly affected by the desorption of O_2_ and N_2_ [63].

### 4.3. Chemical Methods

#### 4.3.1. Amine Scrubbing

Chemical upgrading, and in particular amine scrubbing, is a promising, highly efficient and mature method for converting raw biogas into a natural renewable gas. The method has been reported to be the second most popular technology in the landscape of biogas upgrading [28]. Amine scrubbing involves the use of reactive chemical solvents to capture CO_2_ and H_2_S from biogas and produce high-grade CH_4_ gas exceeding 99% purity. Amines are organic substances obtained from NH_3_ by substituting hydrogen atoms with an alkyl or aromatic group. They covalently bind to acidic gases (e.g., CO_2_ and H_2_S) in a heat-releasing reaction [64,65,66]. Some authors reports on a wide range of organic solvents that can purify CO_2_ and H_2_S from biogas. Commercially available solvents include N-formyl-morpholine, N-methyl pyrrolidone, methanol, and polyethylene glycol ether [67,68].

Bas et al. [69] investigated the use of two aqueous solvents, i.e., mono-ethanolamine (MEA) and N-methyldiethanolamine, to absorb and purify raw biogas from a wastewater treatment plant. MEA was observed to be the most effective absorbent at a 20% solution concentration. At this concentration, the CO_2_ recovery and CH_4_ content were 94% and 96%, respectively. In a similar study, MEA was reported to be the most effective among the four tested amine solvents to upgrade biogas from cow manure, achieving the highest CO_2_ removal rate and increasing CH_4_ content by 19.9% [70]. Using ChemCad 6.3^®^ simulations, MEA was found to be a better amine solution for chemical scrubbing of biogas compared to NaOH and KOH [13].

**Figure 10:**
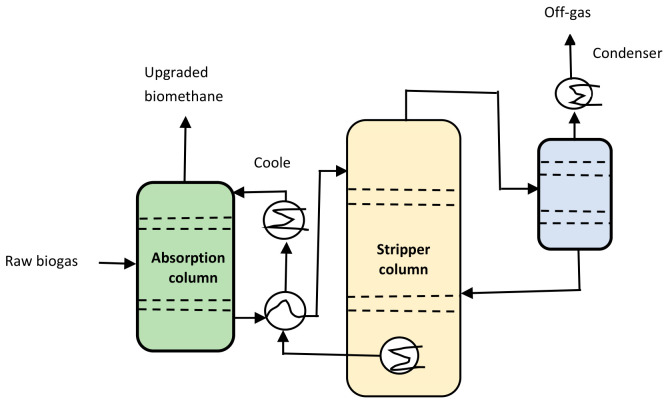
Schematic diagram of amine scrubbing [33].

An amine scrubber is a multi-component unit (Figure 10). It consists of an absorber column, a heat exchanger, a stripper, a condenser and a re-boiler [71,72]. The re-boiler serves to increase the temperature of the inlet amine solvent and to vaporize the CO_2_. Biogas is fed into the absorber by means of an underneath blower. The amine solution is introduced at the top of the absorber. The principle is to purify biogas using a counter flow mechanism. Raw biogas moves up the column while the amine solution flows down the system. This results in a rich amine solution at the bottom of the column, saturated by CO_2_ and H_2_S. The purified CH_4_ is released at the top of the absorber and compressed for further use. The stripper plays a key role as a regeneration column to heat up the rich amine solution in this amine-based gas treatment system. Some CO_2_ that remains in the lean amine solution is vaporized and condensed to remove water that is returned back to the absorber.

The major advantages of amine scrubbing include low CH_4_ spillage (<0.1%) and the ability to recycle the CO_2_ from the amine scrubber [71,72]. However, amine scrubbing consumes large amounts of heat energy (0.40–0.75 kWh/Nm^3^) to regenerate the chemical solvent. It is important to acknowledge that the aqueous amine is highly volatile, which leads to reactant loss and release of toxic gases. Therefore, it is vital to pre-treat raw biogas to compensate for the high energy demand associated with downstream upgrading processes [64,68].

#### 4.3.2. Hydrogenation/Sabatier Reaction

As shown in Equation (3), the Sabatier reaction involves the catalytic reduction of CO_2_ in the presence of H_2_ to generate CH_4_ [73]. This hydrogenation reaction requires the addition of ruthenium or nickel-based catalyst and/or microbial catalysts in a temperature-controlled environment. The Sabatier reaction is reversible, highly exothermic, and thermodynamically unstable. High temperatures, the presence of water, and oxidative conditions often cause deactivation of the nickel-based catalysts, thereby compromising the efficiency and economic viability of CO_2_ methanation via the Sabatier process. This necessitates continual removal of heat from the system for efficient CO_2_ conversion. Ruthenium is somewhat a more sophisticated but expensive catalyst. Current research focuses on developing catalysts that exhibit improved activity at low temperatures. The Sabatier reaction is important to both biological and thermochemical processes deployed in advanced biogas upgrading systems [74,75]. During biogas upgrading, Zhuang and Simakov [76] achieved the highest CO_2_ conversion rate of 91% and 100% selectivity for CH_4_ production in a thermocatalytic Sabatier reactor.

(3) $${CO}_{2}+{4H}_{2}\to {CH}_{4}+{2H}_{2}O$$

### 4.4. Summary of Technologies for Small-Scale Domestic Biogas Upgrading

This paper does not seek to compare biogas upgrading methods or identify the best-fit option, but rather to provide a comprehensive synthesis of small-scale viable options, with emphasis on their utility in decentralized and domestic systems. However, it remains important to present the most promising methods, while acknowledging the techno-economic constraints that may limit their applicability in decentralized contexts. The techno-economic characteristics of biogas upgrading technologies for domestic-scale applications are provided in Table 3. It is important to highlight that conventional biogas upgrading remains the most frequently utilized technology, constituting ~99% of the total upgrading facilities [2].

Small-scale domestic biogas upgrading should balance process simplicity, minimized capital cost, and operational robustness while accomplishing sufficient CH_4_ enrichment for household energy applications. Amongst the available options, the most technically feasible routes for decentralized systems include conventional methods such as pressure swing adsorption, membrane separation, and water scrubbing. However, domestic-scale deployment of these methods is limited by high capital costs of approximately US$ 0.14–0.29 per Nm^3^ of upgraded biogas [77]. More so, high electricity consumption exceeding 0.31 kWh/Nm^3^ constrains the applicability of conventional upgrading at household level [32].

Emerging strategies, including cryogenic separation, biological upgrading, and hybrid technologies, are mostly considered to be more economically viable routes for biogas upgrading. These methods are still undergoing research and development and their technical viability needs to be further improved. There is lack of aggregated cost data for these biogas upgrading technologies. Yet cryogenic separation remains a costly process, with upgrading costs of around US$ 0.51 per Nm^3^ of upgraded biogas. Although emerging technologies are generally reported to be low-cost and environmentally friendly, with low energy needs, their economic performance remains inadequately quantified. In addition, these technologies are better suited to domestic applications and are often not readily scalable to large-scale operations [78].

## 5. Process Control Strategies for Biogas Upgrading

AD systems generally depends on a number of operational parameters, including temperature, pH, mixing regime, feedstock composition and load, etc. Variations in these process conditions significantly affect biogas yield and its composition. Consequently, these changes influence the performance of the biogas upgrading process and the quality of the resulting biomethane [63]. Therefore, the development of process monitoring and control strategies for biogas upgrading systems is fundamental for producing biomethane that can compete with natural gas in the global market. Monitoring and control play a crucial role in ensuring the efficiency, stability, and reliability of biogas upgrading systems. Effective control of biogas purification helps regulate process conditions and ensures the production of biomethane with the desired quality [16].

Gas composition monitoring is a key control strategy in biogas upgrading processes. Monitoring gas composition enables operators to identify process deviations and modify operating conditions to maintain high CH_4_ purity [79]. Gas chromatography (GC) is routinely used to analyze gas samples off-line in a laboratory. However, off-line gas monitoring is often time-consuming and laborious. On-line gas analyzers have been recently developed to continuously measure CH_4_, CO_2_, H_2_S, and O_2_ concentrations in the gas stream. Infrared analyzers are on-line devices designed to detect CH_4_ and CO_2_ while electrochemical sensors are suitable for measuring H_2_ and H_2_S. A µ-GC has been reported to analyze gas composition on-line; thus, it is more appropriate for field measurements than the ordinary GC [17,18].

Pressure exerts a positive impact on both the AD and the CH_4_ recovery process. The operating pressure for conventional biogas upgrading systems such as water scrubbing, physical scrubbing, chemical scrubbing, pressure swing adsorption, and membrane technology typically ranges from 100 to 1200 kPa [27,67]. In 2011, Lindeboom designed a high-pressure AD (HPAD) system to treat different organic substrates by integrating the AD process with in situ high pressure. This innovative technology was primarily designed to generate high-pressure conditions in the digester headspace, thereby synchronizing the pressurization and purification of biogas. The HPAD attained a low CO_2_ footprint of about 13 kg CO_2_/MW h_f_ and CH_4_ purity of up to 99% [80]. Similarly, Scamardella et al. [81] simulated and optimized pressured AD (PAD) technology (150–500 kPa) using Aspen Plus^®^ to directly generate biogas and upgrade it to CH_4_ of high purity (≥95%), without the compression phase. A positive linear relationship between operating pressure and CH_4_ concentration in the biogas stream was reported. Pressure control is essential in upgrading technologies such as membrane separation, pressure swing adsorption, and water scrubbing. Maintaining the required pressure difference improves the separation efficiency between CH_4_ and CO_2_ and increases CH_4_ production [82]. Pressure is usually monitored by means of sensors, transmitters, and gauges, often coupled with automated control systems to maintain the desired operational pressure of 300–1000 kPa, particularly in pressure swing adsorption systems [83].

Temperature is considered a key environmental parameter in most biogas upgrading systems. It can significantly affect gas solubility, the ionic equilibria, and the pH during gas purification. As a result, temperature control is necessary for maintaining operating conditions at a steady state. Many biogas upgrading systems operate within specific temperature ranges to ensure efficient gas separation and stable system performance. According to Agarwal and Singh [84], the most ideal temperature for converting CO_2_ to CH_4_ via catalytic CO_2_ methanation ranges from 300 to 400 °C. Most biological upgrading systems typically operate within a temperature range of 37 to 55 °C [27]. In photobioreactor systems, temperature should be maintained in the range of 15–35 °C to promote microalgal growth [63]. Temperature sensors and controllers are frequently installed in biogas upgrading structures to maintain optimal process conditions [85].

Flow rate control regulates the movement of gas and absorbent liquids within the biogas upgrading infrastructure. Flow meters and automated valves help maintain steady gas and liquid flow rates, which enhances the efficiency of CO_2_ and H_2_S removal [79]. For example, Rodero et al. [63] designed a process control system to monitor biogas flowrate changes in photosynthetic biogas purification. This system was able to maintain CO_2_, O_2_ and H_2_S at concentrations of ≤2.5%, ≤1% and non-detectable limit, respectively. In addition, the resulting CH_4_ content in the purified gas was found to be more than 94%.

In biological upgrading systems, pH should be regularly monitored to maintain stability and suitable conditions for microbial activity. The operating pH for biological biogas upgrading processes, including biomethanation and hydrogen-assisted upgrading, generally ranges from 7.0 to 8.5 [56]. Maintaining an optimal pH range supports the activity of microorganisms responsible for converting CO_2_ into CH_4_ [85]. Also, pH is well-known to influence the dissolution of CO_2_ and H_2_S in biogas upgrading systems. Therefore, variabilities in pH may alter the composition of gas [17]. The rise in pH conditions affects CO_2_ and H_2_S recovery by increasing their solubility in biogas absorption systems [63]. Both real-time and off-line devices are available in the market to control pH fluctuations in AD systems. The process involves immersing calibrated, high-stability pH electrodes in the aqueous scrubbing medium or in the reactor slurry [86].

Sophisticated biogas upgrading plants are now increasingly using automation and feedback control systems. Programmable logic controllers (PLCs) and digital monitoring systems continuously track key variables such as pressure, temperature, and gas composition. These systems automatically adjust process variables to maintain steady operation and improve the overall performance efficiency [82]. An example is remote monitoring via Internet of Things (IoT) technologies, which can be integrated with machine learning tools to measure real-time pressure and flow data in digester systems [16]. According to Mala et al. [20], the emergency of advanced data-driven soft-sensing approaches, particularly artificial intelligence (AI) models, has proven to be an accurate and efficient method for optimizing, scaling up and facilitating the commercialization of gas–liquid membrane contactors (GLMCs) in biogas upgrading systems. These models permit real-time prediction of key process variables, improve control accuracy, and validate improved decision-making in complex upgrading applications.

Overall, effective process control strategies improve CH_4_ purity, increase system stability, and enhance the operational performance of biogas upgrading technologies. Moreover, the development of modernized monitoring systems, automation, and adaptive control mechanisms ensures real-time optimization of process conditions, in so doing regulating process fluctuations and energy consumption. This not only enables consistent CH_4_ quality that meets desired standards, but increases the lifespan of upgrading tools, lowers upgrading costs, and enhances the overall sustainability and reliability of biogas purification systems.

## 6. Domestic Applications of Biomethane

Biomethane is a renewable upgraded form of biogas with a high CH_4_ concentration. It is the most valuable component of biogas and possesses several unique opportunities for domestic infrastructure. This is due the fact that its properties are analogous to those of standard natural gas. The consumption of biomethane at household level aligns with the United Nations Sustainable Development Goals, a blueprint proposed to ensure clean and affordable energy access, reduced reliance on fossil fuels and climate change, and improved health service delivery [87]. The use of purified biogas incorporates biomass resources and sustainable technologies, offering holistic environmental benefits that support a circular economy. These benefits are six-fold: (1) it is a renewable power source; (2) it can replace petroleum-based fuels; (3) it can combat the release of CH_4_ into the atmosphere; (4) it can lower the CO_2_ emission from combustion; (5) it can be potentially utilized in all natural gas appliances; and (6) it can address challenges of waste management [14,33]. Some of the potential domestic applications of biomethane include cooking, space and water heating, lighting, and power generation, amongst others [26].

An important consideration in decentralized biogas systems is the linkage between gas quality and the performance requirements of domestic end-user appliances. Although standards such as EN 16723 are aimed for grid injection and transportation, their specifications offer a relevant framework for defining minimum gas quality thresholds for decentralized systems. Accordingly, maintaining CH_4_ concentrations above 90% and reducing impurities to trace levels are important to ensure compatibility with domestic-scale appliances [12]. This represents a feasible approach for improving safety, reliability, and efficiency of small-scale biogas systems.

Household cooking is one of the most popular domestic applications of biomethane. Biomethane can be supplied via small-scale digesters or stored in cylinders to fuel gas stoves for use in food preparation [88]. Biogas stoves have a simple design consisting of a metal base and body. They are relatively cheap, although their flame spreader requires corrosion-resistant metals [26]. Compared to conventional fuel sources like fuelwood, charcoal, and paraffin, biomethane combusts more efficiently, emits less fumes, and mitigates indoor air pollution.

Biomethane is a suitable fuel for residential heating systems such as water and space heating [88]. It can be utilized in gas water heaters to provide hot water for household activities. Hot water can be used for bathing, washing dishes, and other sundry applications. Biomethane provides a reliable and efficient heat source for homes and offices. It is also a source of fuel for space heating in cooler regions or during cold months using gas heaters or small household boilers. This helps preserve warm indoor temperatures while reducing reliance on electricity or traditional fuels [88]. In agriculture, the use of radiant heaters is quite a common practice in animal rearing. These infrared heaters are made up of clay materials and fitted with air conditioners. They can be warmed up with biomethane to high temperatures of around 600–800 °C. However, these types of heaters need proper handling and servicing [89].

Nowadays, biogas lamps are designed to burn biomethane as a fuel. These lamps provide lighting in pastoral or off-grid households, where access to electricity is limited. This alleviates the dependence on petroleum fuels, which is the primary source of energy for electric power generation throughout the world. Nonetheless, there is limited use of biogas lamps, mainly due to low combustion efficiency, which can often result in high heat or even fire [26].

Biomethane can be used in power absorption refrigerators, which function using heat from gas combustion instead of electricity. This application is typically useful for food preservation in off-grid households. An absorption refrigerator is generally a type of fridge that uses NH_3_ and water to drive the cooling process. It is highly recommended to use a safety pilot when using biogas as a fuel to power this fridge. A 0.1 m^3^ absorption fridge can consume about 2 m^3^ of biogas per day. This consumption is significantly influenced by the ambient temperature conditions [62].

Upgraded biogas from biomass resources is an economical source of fuel for heat and electricity generation in domestic settings. Biomethane can be used to operate small gas generators to produce household electricity for basic needs such as lighting, charging devices, and powering small appliances. This is particularly useful in remote rural communities with lack of access to grid power. Developing regions like Asia, Africa and South America have promoted this initiative through funding and incentives from governments and non-governmental institutions [88]. Biogas can be utilized for electricity generation via combined heat and power (CHP) systems using boilers and gas engines. In such systems, the device is designed to condense water vapor in biogas and prevent condensation within the gas delivery pipelines, which could otherwise cause operational problems. Although CHP turbines are capable of operating with relatively low-purity biogas, the concentration of H_2_S must typically be maintained below 250 ppm to prevent corrosion and rusting of boilers, engines, and associated components. Despite these advantages, one of the main limitations of using biogas for CHP generation is its relatively low calorific value. This reduces the overall energy output compared to traditional fossil fuels [33].

In past decades, the use of biogas to offset natural gas has significantly increased due to shortage and poor quality of the petroleum gas. Upgraded biogas lines can be connected to the natural gas grids. This has been reported in countries such as Sweden, Germany, Switzerland and France, where the biogas upgrading technologies meet the standards of injection into the natural gas grid [33]. In the developing world where infrastructure exists, purified biogas can be fed into local gas distribution networks, serving homes for cooking, heating, and other domestic energy requirements.

It cannot be overemphasized that biomethane offers a versatile and sustainable energy solution for domestic use, particularly in rural and peri-urban areas. Its adoption can reduce reliance on solid biomass fuels and mitigate energy poverty, climate change and indoor pollution.

## 7. Challenges and Prospects

The demand for biogas and biomethane has globally increased by 4% to around 1.76 EJ in 2023 [90]. Much of the work revolves around Europe, China, the Americas and India, where production systems are fully developed [91]. Upgrading raw biogas into high-quality biomethane suitable for domestic applications represents an important trajectory toward sustainable energy transitions and a circular economy. Despite significant technological advancements, various technical, economic, social, and regulatory challenges continue to distract the widespread adoption of biogas technology [92]. Meanwhile, emerging innovations offer promising prospects to solve these problems and improve system performance, economic feasibility, and scalability.

### 7.1. Feedstock Availability and Process Instability

Biogas composition is inherently affected by feedstock variability and operating variables, often resulting in significant changes in CH_4_ and CO_2_ volumes. This is particularly pronounced in decentralized AD systems, where feedstocks are primarily sourced from household kitchen waste, crop residues, or livestock manure with highly variable composition. These fluctuations often complicate downstream upgrading technologies and compromise process stability [31,93,94]. In small-scale domestic systems, these variations are more critical due to a lack of advanced control mechanisms typically found in commercial plants. For example, in situ biological biogas upgrading inherently suffers from process inhibition, which affects methanogens at pH values exceeding 8.5. This surge in pH is a result of the removal of bicarbonates that provide buffering capacity in AD systems. The oxidation of VFAs and alcohols caused by low volumes of H_2_ (>10 Pa) is also an obstacle to this process. Oxidation often results in accumulation of propionate, lactate, and ethanol that can hinder in situ biological upgrading [2].

Furthermore, unpredictable feedstock production and poor digester performance common in household level systems adversely affect the amount and quality of biogas. This imposes substantial problems for constant biomethane production required to consistently operate domestic energy appliances for cooking and heating [31,92,94]. It is fundamental to integrate advanced technologies such as digitalization and smart process control in biogas upgrading for small-scale and decentralized systems. AI, machine learning and modern sensors allow real-time monitoring and optimization of the purification processes. If properly scaled and economically optimized, these smart tools can predict failures, improve process stability, and increase the CH_4_ output, creating systems ideal for domestic purposes.

### 7.2. High Capital Needs

To some extent, biogas production and purification is a costly business venture that can constrain the long-term sustainably for domestic applications. Sophisticated upgrading technologies such as water scrubbing, chemical absorption, membrane separation, and pressure swing adsorption necessitate the need for large sums of money and high energy. Adsorption processes typically operate at elevated pressures and temperatures, making them inherently energy-demanding and costly processes. This is a key barrier to domestic-scale adoption, particularly in developing regions, where the technologies are immensely required [94]. For practical household applications, biogas upgrading must be simple and affordable and utilize locally available materials.

The intervention of the private sector, as well as governmental and academic institutions, is critical to minimize costs associated with biomethane generation and its distribution to end-users. The integration of several biogas upgrading technologies into hybrid systems necessitates a concerted effort to eliminate weaknesses of individual methods, obtain higher CH_4_ yields, minimize energy use, lower operational costs, and enhance the overall system performance [95]. It is also encouraged to merge biogas upgrading with broader energy platforms to improve resource efficiency and generate extra income. Such technological platforms include the Power-to-X system that turns CO_2_ into high-value fuels like methanol. This strategy is particularly suitable for decentralized facilities where attaining stable and high-quality CH_4_ is critical for reliable household energy needs.

### 7.3. Contaminants and Scrubbing Constraints

Biogas is made up of many contaminants such as CO_2_, H_2_S, NH_3_, H_2_O and siloxanes, which must be removed to produce biomethane that matches the quality of natural gas. In domestic and decentralized systems, the presence of these contaminants is a challenge due to the utilization of cheap resources and simple equipment designs. Failure to adequately remove these impurities will corrode gas equipment, lower heating value, and present potential health threats. These obstacles are mainly linked to the quality standards outlined in the EN 16723 guidelines, which emphasizes strict limits on gas impurities to ensure safety and efficiency in gas appliances. However, attaining such high quality levels in small-scale systems remains difficult.

Khan et al. [2] and Nevzorova and Kutcherov [96] identified clogging of pipelines caused by high volumes of contaminants as well as CH_4_ and CO_2_ losses as the major hurdles to the widespread adoption of new biogas upgrading technologies. Therefore, generating high-quality CH_4_ (>95%) and reducing energy demand is a technical liability [97]. More scientific research is required to improve process efficiency and surpass these barriers, while developing user-friendly upgrading technologies tailored for small-scale biogas systems.

### 7.4. Production of Toxic Compounds

Some authors have reported the release of particulate matter from burning purified biogas. According to Das et al. [31], these toxic gases fall within the ultrafine particle (UFP) spectrum comparable to conventional fuel sources and solid biomass fuels. This may pose significant adverse health and environmental risks. In small-scale rural settings, this problem is particularly disturbing, as cooking and heating usually occur in confined or poorly ventilated conditions, increasing the risk of direct human exposure. Women and children are particularly vulnerable to household air pollution as they spend a disproportionate amount of time near cooking areas where inefficient gas stoves are frequently used [98]. In addition, the deposition of sulfur-containing compounds and siloxanes on burners and other components may damage gas stoves and lower combustion efficiency. Thus, further research is needed to reduce the volumes of sulfur-containing substances and siloxanes in biomethane. This will enable the minimal emission of particulate matter, ensuring it remains within the safe UFP range for reliable and low-emission domestic energy applications.

### 7.5. Energy Consumption and Operational Efficiency

Several biogas upgrading processes consume significant amounts of energy, limiting the total energy productivity of biomethane systems. For example, operations like compression, regeneration of solvents, and vacuum processes raise the overall energy input, thus influencing the sustainability and cost-effectiveness of biogas upgrading systems. The use of devices such as turbines and compressors in cryogenic separation and the regeneration of amine solution in chemical scrubbing require substantial energy inputs. Subsequently, this results in increased operational costs and carbon footprints, thus restraining the overall sustainability and wide-scale acceptance of these processes. This underscores an urgent need to design and implement energy-efficient devices and process configurations for biogas upgrading [99].

### 7.6. Technological Scalability

Most biogas upgrading systems are effective at commercial scale, but somewhat costly and complex. This limits their utility for small-scale or household-level facilities. However, there is a paradigm shift in technological scalability from centralized industrial models toward small-scale decentralized systems suitable for rural homes and small farm settings. Commercial methods (e.g., membrane technology, amine scrubbing, etc.) that produce high-quality biomethane are quite expensive for domestic applications, motivating the search for cheaper substitutes such as lime water scrubbing and charcoal adsorption. Transitioning large-scale methods without compromising efficiency and reliability is a key obstacle, especially for small-scale decentralized applications [100]. Technological advancements should focus on designing compact, modular upgrading units tailored for pastoral and off-grid communities. Such infrastructure can mitigate reliance on consolidated systems, ensuring on-site biogas production and purification for domestic energy requirements.

### 7.7. Policy, Regulatory, and Market Barriers

The market growth of biogas upgrading for smallholder domestic applications is restricted by inconsistent regulatory frameworks, limited biomethane standards, and insufficient incentives [96,101]. In some regions, the incorporation of biomethane into domestic energy systems is hindered by a lack of equipment for gas delivery and packaging. Subsequently, households and domestic users face barriers in accepting upgraded biogas technologies. As such, the widespread interest in renewable fuels and decarbonization is driving policy reforms, subsidies, incentives, and investment initiatives that gradually consider decentralized energy solutions. The demand for biomethane will continue to rise in the foreseeable future, reinforcing its role in small-scale and centralized energy systems. To accelerate domestic-scale adoption, decision-makers should craft policy and regulatory frameworks with the intention to source funds, foster collaborations, and enhance flexibility in the biogas sector.

## 8. Conclusions

The upgrading of raw biogas into high-quality biomethane for domestic applications is essential for advancing sustainable energy systems and mitigating dependency on petroleum fuels. This review emphasizes that whereas traditional upgrading methods, including water scrubbing, chemical absorption, pressure swing adsorption, and membrane separation, have made significant progress, they continue to face challenges due to energy intensity, hefty operational expenses, and vulnerability to contaminants. Cutting-edge technologies such as hybrid systems, biological upgrading, advanced gas separation, and catalytic methanation provide viable solutions to improve process efficiency and biomethane quality while alleviating ecological effects. It is possible that the coupling of these methods, reinforced by process optimization and digital monitoring, offers a promising strategy to enhance overall process performance and financial viability for decentralized small-scale systems. Nevertheless, technological bottlenecks such as scalability, sustainability, and contamination control (e.g., H_2_S and siloxanes) require consideration to ensure consistent performance. The transition from traditional biogas systems to innovative biomethane solutions for household facilities will rely on surpassing existing economic and technical barriers. Emerging research should concentrate on exploring energy-efficient, affordable, and vibrant purification systems tailored for domestic applications, together with supportive policy environments, financial incentives, and standardization. Finally, the use of ground-breaking technologies for biogas upgrading is pivotal for accelerating the shift toward carbon-neutral, readily available, and sustainable energy solutions for household and community-level applications. Recent developments in materials science, biological processes, and digital technologies are revolutionizing the field, enabling system robustness and circular bioenergy solutions.

## Figures and Tables

**Table 1:** Biogas composition [26,28].

Gas Constituent	Composition
Methane	22–70%
Carbon dioxide	35–40%
Nitrogen	0–3%
Hydrogen	0–3%
Oxygen	0–5%
Hydrogen sulfide	0–1%
Siloxanes	0–50 mg/m^3^
Volatile organic compounds	0–4500 mg/m^3^
Halocarbons	20–200 ppm

**Table 2:** Mechanisms, pros and cons of biogas upgrading technologies [5,33].

Method	Mechanism	Pros	Cons
Physical	Water scrubbing	• High CH_4_ content (~99%). • CO_2_ and H_2_S removed concurrently. • Simple and easy with minimized CH_4_ loss. • No use of toxic chemicals.	• Huge capital costs. • High pressure and energy demands. • High water requirements. • High exposure to biological contaminants.
	Pressure swing adsorption	• Low energy and capital needs. • High CH_4_ purity (96–98%). • Safe and simple method. • Water-free method.	• Up to 4% CH_4_ loss. • Require pre-purification to remove H_2_S. • Require pre-drying to remove water. • Biogas impurities may contaminate adsorbents.
	Absorption using amine solutions	• Highly efficient method. • Around 99% CH_4_ purity. • Simple operation. • Low CH_4_ loss (<0.1%). • CO_2_ and H_2_S removed at the same time.	• Huge capital cost for amine solvents. • Toxicity of amine solvents. • Solvent loss due to evaporation. • Huge energy input. • Waste chemical solutions must be safely disposed.
	Adsorption using organic solvents	• High CH_4_ purity (~98%). • Minimized liquid use. • Smaller sizes of the upgrading system. • H_2_S and CO_2_ removed simultaneously. • Simple and easy of operation. • Reduced CH_4_ loss.	• Toxicity of organic solvents. • Challenges in regenerating organic solvents caused by high CO_2_ solubility. • H_2_S removal requires high temperatures.
	Cryogen separation	• Eco-friendly technology • More than 97% CH_4_ purity. • Highly pure CO_2_ recovered.	• High capital and operational costs. • High energy input. • The method is still naïve.
	Membrane separation	• Small space is required. • Easy installation. • Low start-up, operational and energy costs. • Very reliable process. • Simple and environmentally benign.	• High cost and fragility of the membrane. • Multi-membrane steps required to obtain high-purity CH_4_. • May require pre-treatment. • Low membrane selectivity.
Chemical	Hydrogenation/ Sabatier reaction	• Highly selective and efficient process. • Minimizes CO_2_ emission by converting it into CH_4_. • Increased CH_4_ purity.	• Trace impurities in biogas have significant impact on the process. • Large amounts of pure H_2_ gas are required. • Require expensive catalysts. • High energy input.
Biological	Photosynthetic reaction	• Up to 97% CH_4_ recovery. • CO_2_ converted into useful products. • Active biomass is produced. • Minimized land and water requirements.	• High capital and energy costs. • Reduced photosynthetic CO_2_ input. • High natural resource use. • Exposure to biological contaminants is high.
	Chemoautotrophic reaction	• High selectivity and process efficiency. • CO_2_ emission reduced by conversion into CH_4_. • Mild temperature and pressure conditions. • It can be easily merged with the AD process. • Eco-friendly process.	• Requires large amounts of reductant. • The method is still at its infant stage.

**Table 3:** Techno-economics of biogas upgrading for domestic-scale application [27,67,77].

Method	Upgrading Cost Per Nm^3^/Biogas Upgraded (US$)	Electricity Consumption (kWh/Nm^3^)
Water scrubbing	0.15	0.45
Pressure swing adsorption	0.29	0.45
Membrane separation	0.14–0.25	0.25–0.43
Cryogenic separation	0.51	0.51
Biological upgrading	-	-
Hybrid methods	-	-

## Data Availability

No new data were created or analyzed in this study.

## Abbreviations

The following abbreviations are used in this manuscript:

AD	Anaerobic digestion
AI	Artificial intelligence
CHP	Combined heat and power
GC	Gas chromatography
GLMCs	Gas–liquid membrane contactors
HPAD	High-pressure anaerobic digestion
IoT	Internet of Things
MEA	Mono-ethanolamine
PAD	Pressured anaerobic digestion
PAT	Process analytical technology
PLCs	Programmable logic controllers
SOB	Sulfate-oxidizing bacteria
UFP	Ultrafine particle
